# Attention‐deficit/hyperactivity disorder symptoms and dietary habits in adulthood: A large population‐based twin study in Sweden

**DOI:** 10.1002/ajmg.b.32825

**Published:** 2020-10-07

**Authors:** Lin Li, Mark J. Taylor, Katarina Bälter, Ralf Kuja‐Halkola, Qi Chen, Tor‐Arne Hegvik, Ashley E. Tate, Zheng Chang, Alejandro Arias‐Vásquez, Catharina A. Hartman, Henrik Larsson

**Affiliations:** ^1^ School of Medical Sciences Örebro University Örebro Sweden; ^2^ Department of Medical Epidemiology and Biostatistics Karolinska Institutet Stockholm Sweden; ^3^ Public Health Sciences Mälardalen University Västerås Sweden; ^4^ Department of Biomedicine University of Bergen Bergen Norway; ^5^ The Department of Psychiatry & Human Genetics Donders Institute for Brain, Cognition, and Behavior, Radboud University Medical Center Nijmegen Netherlands; ^6^ Department of Psychiatry University of Groningen, University Medical Center Groningen Groningen Netherlands

**Keywords:** ADHD, adults, dietary habits, genetic correlation, phenotypic correlation, twin study

## Abstract

Associations between adult attention‐deficit/hyperactivity disorder (ADHD) symptoms and dietary habits have not been well established and the underlying mechanisms remain unclear. We explored these associations using a Swedish population‐based twin study with 17,999 individuals aged 20–47 years. We estimated correlations between inattention and hyperactivity/impulsivity with dietary habits and fitted twin models to determine the genetic and environmental contributions. Dietary habits were defined as (a) consumption of food groups, (b) consumption of food items rich in particular macronutrients, and (c) healthy and unhealthy dietary patterns. At the phenotypic level, inattention was positively correlated with seafood, high‐fat, high‐sugar, high‐protein food consumptions, and unhealthy dietary pattern, with correlation coefficients ranging from 0.03 (95%CI: 0.01, 0.05) to 0.13 (95% CI: 0.11, 0.15). Inattention was negatively correlated with fruits, vegetables consumptions and healthy dietary pattern, with correlation coefficients ranging from −0.06 (95%CI: −0.08, −0.04) to −0.07 (95%CI: −0.09, −0.05). Hyperactivity/impulsivity and dietary habits showed similar but weaker patterns compared to inattention. All associations remained stable across age, sex and socioeconomic status. Nonshared environmental effects contributed substantially to the correlations of inattention (56–60%) and hyperactivity/impulsivity (63–80%) with dietary habits. The highest and lowest genetic correlations were between inattention and high‐sugar food (*r*_A_ = .16, 95% CI: 0.07, 0.25), and between hyperactivity/impulsivity and unhealthy dietary pattern (*r*_A_ = .05, 95% CI: −0.05, 0.14), respectively. We found phenotypic and etiological overlap between ADHD and dietary habits, although these associations were weak. Our findings contribute to a better understanding of common etiological pathways between ADHD symptoms and various dietary habits.

## 1 INTRODUCTION

Attention‐deficit/hyperactivity disorder (ADHD) is a neurodevelopmental disorder with an approximate prevalence of 5% in children and adolescents and 2.5% in adults (Stephen V. Faraone et al., 2015; Sayal, Prasad, Daley, Ford, & Coghill, 2018; Simon, Czobor, Balint, Meszaros, & Bitter, 2009). ADHD is associated with many adverse outcomes across development (Asherson, Buitelaar, Faraone, & Rohde, 2016; Stephen V. Faraone et al., 2015). The pathophysiological mechanisms underlying ADHD are unclear and its diagnostics and treatment remain challenging (Sharma & Couture, 2014).

Dietary habits, a modifiable lifestyle factor, play a fundamental role in brain development, physiology, and functioning (Brandt, 2019). In the two most recent systematic review and meta‐analyses, ADHD was reported to be positively associated with unhealthy dietary habits (characterized by high consumption of refined sugar and saturated fat), and negatively associated with healthy dietary habits (high consumption of fruits and vegetables) among children and adolescents (Del‐Ponte, Quinte, Cruz, Grellert, & Santos, 2019; Shareghfarid, Sangsefidi, Salehi‐Abargouei, & Hosseinzadeh, 2020). However, only two available studies with adult samples were found with conflicting results. Weissenberger et al. (2018) reported an association between ADHD symptoms and higher consumption of sweets, while Holton, Johnstone, Brandley, and Nigg (2019) found nutrient intake was not associated with diagnosed ADHD.

Due to the lack of evidence from longitudinal studies, previous studies have failed to explain the exact direction of the associations and underlying mechanisms. Diet has been suggested as a potential intervention or treatment of ADHD for many years. According to double‐blind placebo‐controlled evidence, a few‐foods diet (a diet based on rice, lamb, lettuce, pears, and water) seems to be the most promising dietary interventions for a reduction in ADHD symptoms in children (Pelsser, Frankena, Toorman, & Rodrigues Pereira, 2017). Although the underlying biological basis remains unclear, recent gut‐brain axis research has shown that human gut microbiota responds rapidly to dietary changes and produces neurochemicals comparable to those produced by the brain (David et al., 2014; Lyte, 2013; Suez et al., 2014). On the other hand, twin studies have suggested that the two core symptom domains of ADHD‐inattention and hyperactivity/impulsivity‐ share genetic factors, but also that the two dimensions also have significant unique genetic underpinnings (Greven, Rijsdijk, & Plomin, 2011; McLoughlin, Ronald, Kuntsi, Asherson, & Plomin, 2007). Therefore, it is possible that the two subdomains of ADHD may contribute to having an unhealthy or less‐balanced dietary habit via separated, but related, genetic factors (Samuele Cortese et al., 2008; Samuele Cortese et al., 2015; Nigg et al., 2016). The core symptoms of inattention, poor planning, and self‐regulation deficits, may cause difficulties in adhering to a regular eating pattern, favoring abnormal eating behaviors (Samuele Cortese et al., 2008; Nigg et al., 2016). In contrast, deficient inhibitory control and delay aversion, which are expressions of the hyperactivity/impulsivity component of ADHD may translate into impulsive eating of highly palatable foods or having no patience to eat vegetables, which are less rewarding than high‐caloric foods (Samuele Cortese et al., 2008; Mian et al., 2019; Nigg et al., 2016). However, none of the previous studies on children, adolescents, and adults has distinguished between the inattentive and hyperactivity/impulsivity components of ADHD. Consequently, it is not clear whether the dimensions of ADHD may specifically be associated with various dietary habits (Samuele Cortese et al., 2008; Nigg et al., 2016).

An important alternative explanation of the association between ADHD symptoms and dietary habits is that these two traits may share some etiological factors. ADHD is strongly influenced by genetic factors, with heritability estimates (ℎ^2^) between 70 and 80% in both children and adults (Asherson & Gurling, 2012; S. V. Faraone et al., 2005; Larsson, Chang, D'Onofrio, & Lichtenstein, 2014), and there are strong genetic links between ADHD diagnoses and sub‐threshold variations in ADHD traits, thus supporting a dimensional model of ADHD (Larsson, Anckarsater, Rastam, Chang, & Lichtenstein, 2012; Taylor et al., 2019). Diet composition is also reported to be moderately heritable, with *h* ^2^ estimates between 27 and 70% (Hasselbalch, Heitmann, Kyvik, & Sorensen, 2008; Meddens et al., 2018; Wade, Milner, & Krondl, 1981). Emerging support for the hypothesis that these two traits may share some etiological factors has been provided by a recent genome‐wide association study of ADHD that reported significant genetic correlations between ADHD and several metabolic traits (genetic correlation, *r*_g_ = .22–.30) (Demontis et al., 2018). An advanced understanding of the associations between ADHD and dietary habits has important clinical and public health implications, because dietary habits may also explain the link between ADHD to health‐related outcomes such as metabolic syndromes (e.g., obesity)(Samuele Cortese et al., 2015; S. Cortese & Tessari, 2017).

The aims of this study were three‐fold. First, we aimed to identify and quantify the associations between ADHD symptom dimensions and different dietary habits in adults. Second, given that ADHD was more common among young people, males and those with socioeconomic disadvantages (Stephen V. Faraone et al., 2015; Rowland et al., 2018), and healthy dietary habits were also reported to be related to age, sex, and SES (Johansson, Thelle, Solvoll, Bjørneboe, & Drevon, 1999), we further explored the association patterns among different age, sex, and SES groups. Third, we aimed to investigate the relative contribution of genetic and environmental factors to the associations between adult ADHD symptom dimensions and different dietary habits, and test for a causal association between these phenotypes by using a large population‐based twin sample in Sweden.

## 2 METHODS AND MATERIALS

### 2.1 Participants

In May 2005, a total of 42,582 Swedish twins born between 1959 and 1985 who survived their first birthday, were identified from the population based Swedish Twin Register (Lichtenstein et al., 2006). Of the target population, 25,364 (59.6%) individuals participated in the Study of Twin Adults: Genes and Environment (STAGE). Participants were given a web‐based survey, which included 1,300 questions, in 34 sections, regarding lifestyle, mental, and physical health. Up to three reminders were sent out to nonresponders, and participants were offered a telephone interview if they preferred this over the web‐based survey. Questionnaire data and age information were available for 24,872 individuals. The response rate for ADHD symptoms and Food Frequency Questionnaire (FFQ) was 71.84% (*n* = 17,867) and 36.8% (*n* = 9,156), respectively. This resulted in 17,999 individuals who provided information about either ADHD symptoms and/or FFQ, who were included in the analyses (Figure S1). Twins who responded to the FFQ questionnaire were more likely to be female (53.83%), have lower SES (50.26%) and higher ADHD symptoms scores compared to nonrespondents. No statistically significant differences were found across age and zygosity groups (Table S1).

All participants provided informed consent. The project has been reviewed and approved by the Regional Ethics Committee at the Karolinska Institutet, Stockholm, Sweden (DNR: 03–224).

### 2.2 Measures

#### 2.2.1 ADHD symptoms

Self‐reported ADHD symptoms were obtained via nine inattention items and nine hyperactivity/impulsivity items covering the 18 DSM‐IV symptoms of ADHD (Larsson et al., 2013). Each item had a three‐point answer format (0 = “No,” 1=“Yes, to some extent,” 2 = “Yes”). The items were summed up to create two subscales of ADHD symptoms‐inattention and hyperactivity/impulsivity. Both inattention and hyperactivity/impulsivity showed good internal consistency, and Cronbach's *α* was .79 and 0.77, respectively. A validation study on our ADHD instrument can be found elsewhere (Larsson et al., 2013).

#### 2.2.2 Dietary habits

Habitual dietary intake was assessed using a food frequency questionnaire (FFQ), comprising of 94 food items. For each food item, the participants indicated their average consumption frequency over the past year by selecting one of the following frequency categories: never, 1–3 times/month, 1–2 times/week, 3–4 times/week, 5–6 times/week, 1 time/day, 2 times/day, 3 times/day. The frequency for each food item was converted into number of servings per day. Dietary habits were expressed in three ways (a) consumption of food groups (fruits, vegetables, dairy, meat, and seafood), (b) consumption of food items rich in a particular macronutrient (high‐fat food, high‐carbohydrate food, high‐sugar food, and high‐protein food), and (c) healthy dietary patterns (fruits, vegetables, fish, and white meat) and unhealthy dietary patterns (food with high proportions of refined sugar and saturated fat) respectively, in line with previous studies on the associations between ADHD and dietary (Del‐Ponte, Quinte, Cruz, Grellert, & Santos, 2019). Detailed information about the definitions of dietary habits can be found in Table S2. The scores of each dietary habit were calculated as the total daily intake frequency of each food group.

#### 2.2.3 Sociodemographic measures

Participants were between 20 and 47 years of age at the time of assessment and categorized into three groups (20–29, 30–39, and 40–47 years). Socioeconomic status (SES; Larsson, Dilshad, Lichtenstein, & Barker, 2011) of the participants was indicated by their occupational status: (I) unskilled and semiskilled workers, (II) skilled workers/assistant nonmanual employees, (III) intermediate nonmanual collar workers, and (IV) employed and self‐employed professionals, higher civil servants, and executives. Higher SES was defined as those with occupational status in Class II, III, or IV.

#### 2.2.4 Zygosity

Standard physical similarity questions that have previously been validated through genotyping were used to establish zygosity. Individuals from both complete (*n* = 10,876) and incomplete (*n* = 7,123) twin pairs were included in the twin analyses.

### 2.3 Data analysis

The distributions of all main variables were positively skewed (range of skewness: 0.84–4.56) and therefore log‐transformed before analysis (range of transformed skewness: −0.70 to 0.64) (Table S3).

### 2.4 Phenotypic analyses

At the phenotypic level, mean differences in ADHD symptoms and dietary intake across sex, age, and SES groups were estimated using linear mixed effect models in SAS version 9.4 (SAS Institute Inc., Cary, NC), which allowed us to account for the dependent nature of the twin observations. Partial pairwise Pearson's correlation coefficients were estimated to quantify the associations between ADHD symptoms and different diets. As the two core symptom dimensions of ADHD, inattention, and hyperactivity/impulsivity, were highly correlated with each other, we further tested whether the correlations between one ADHD symptom dimension and dietary habits could be ascribed to the other ADHD symptom dimension. To test the association patterns among different age, sex, and SES groups, we further conducted exploratory analyses by stratifying on these factors.

### 2.5 Genetically informative analyses

The twin method relies on the different levels of genetic relatedness between monozygotic twins (MZ), who are genetically identical, and dizygotic twins (DZ), who share on average 50% of their polymorphic genetic variation. This information was used to decompose the variance of each phenotype and the covariation between phenotypes into additive genetic factors (A), dominant genetic factors (D), environmental factors shared by twin pairs (C) and nonshared or unique environmental factors (E), including measurement error (Rijsdijk & Sham, 2002). As the C and D components cannot be estimated in the same model using the classical twin design, we fitted ADE and ACE models separately and then compared goodness‐of‐fit of the two models. A greater intraclass correlation (ICC) is expected in MZ twins than in DZ twins if there are genetic influences on a trait. Similarly, in the cross‐twin cross‐trait correlations (CTCT), where we estimated the correlations of ADHD symptoms of twin 1 and the dietary habits of twin 2, higher CTCT in MZ twins than in DZ twins indicate genetic influences on the covariation between traits.

Structural equation modeling in the statistical software R version 3.6.1, with the OpenMx package (2.14.11), was used to conduct univariate and bivariate analysis based on raw data (Neale et al., 2016). Maximum‐likelihood model‐fitting, which allowed handling of missing data and inclusion of incomplete twin pairs in models, was performed on each phenotype. In the univariate and bivariate analyses, we first fitted a fully saturated model to estimate the means, variances, and covariances in observed data. Several sub‐models were fitted to test assumptions (equating means and variances across twin order, zygosity, and sex) by performing likelihood ratio tests comparing the current nested model with the previous best‐fitting model. Akaike Information Criterion (AIC) was additionally used to assess the fit of each solution. In the bivariate models, including one ADHD symptom dimension (inattention or hyperactivity/impulsivity) and one dimension of diets, we choose to fit an ADE model based on the observation that ICC and CTCT correlations in MZ twins were consistently more than twice the size of those in DZ twins. Two sub‐models (AE and E models) were fitted to evaluate whether they would explain the data significantly worse than ADE model and test the presence of D and A. The bivariate models also provide estimates of additive genetic correlation (*r*_A_), dominance genetic (*r*_D_) and nonshared environmental (*r*_E_) correlations, which vary from −1.0 to +1.0. These statistics indicate to what extent genetic and environmental influences in one phenotype overlap with the other.

To further test the robustness of the nonfamilial overlap and the causal associations between ADHD symptoms and dietary habits, we computed the differences of the two ADHD symptom dimensions between an MZ twin and his/her co‐twin. These intrapair differences in symptoms were then regressed on the MZ twin differences in dietary habits. Statistically significant and positive associations (i.e., the MZ twin with more ADHD symptoms than his/her co‐twin also consumes more unhealthy or high‐sugar food than his/her co‐twin) would point toward a causal effect.

## 3 RESULTS

### 3.1 Phenotypic associations

**FIGURE 1:**
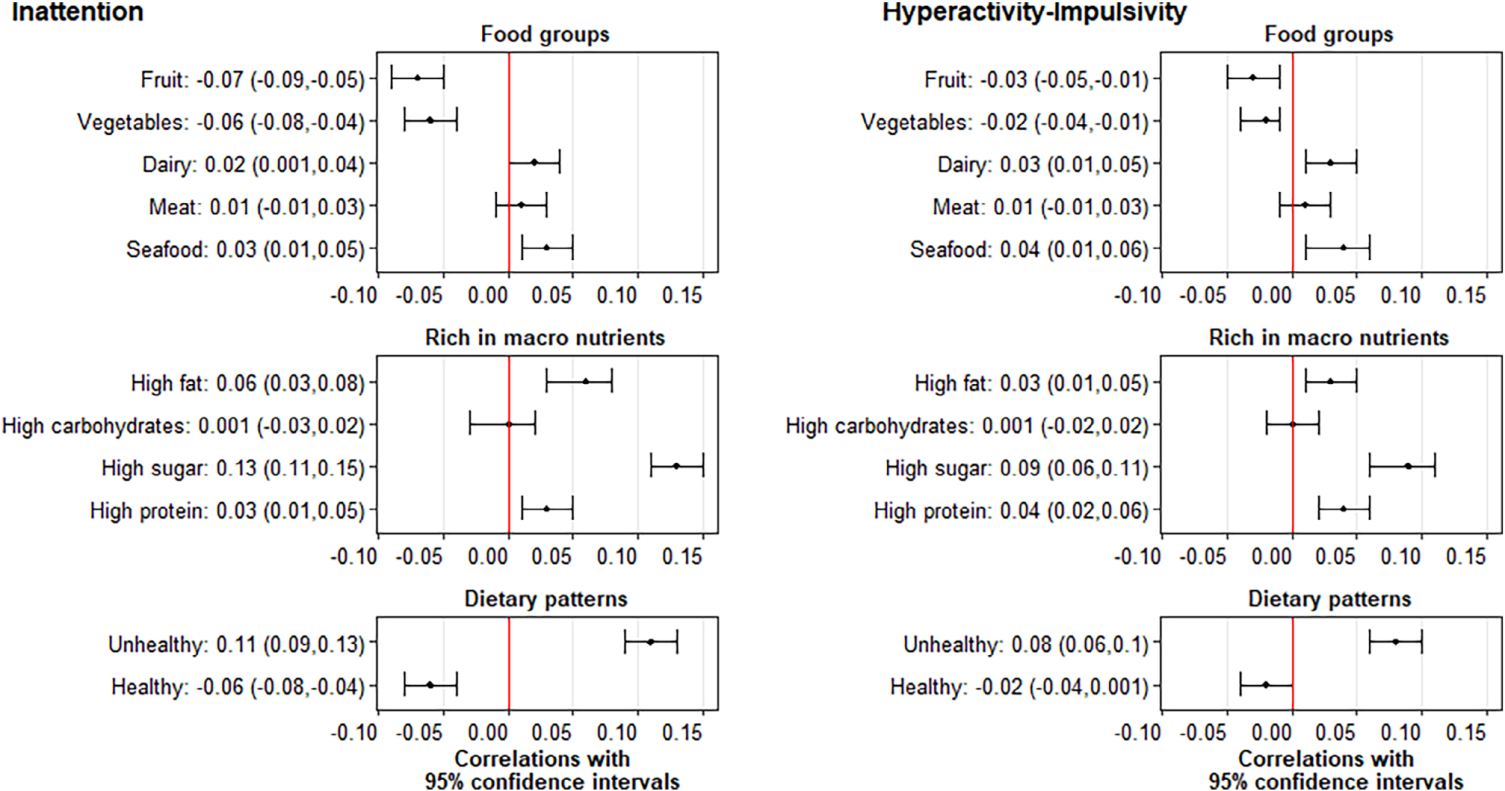
The correlations with 95% confidence intervals between ADHD trait dimensions and different dietary habits (adjusted the relatedness of individuals by using Partial pairwise Pearson's correlation analysis), *N* = 8,731 [Color figure can be viewed at wileyonlinelibrary.com]

Descriptive statistics are presented in Table 1. Figure 1 shows the correlations between ADHD symptom dimensions and different dietary habits. Inattention was positively associated with high consumption of seafood, high‐fat food, high‐sugar food, high‐protein food, and unhealthy food, with correlations ranging from 0.03 (95% CI: 0.01, 0.05) for seafood to 0.13 (95% CI: 0.11, 0.15) for high‐sugar food. There were negative correlations between inattention and fruits, vegetables, and healthy food intake; the range of the correlations was from −0.06 (95% CI: −0.08, −0.04) for vegetables and healthy food to −0.07 (95% CI: −0.09, −0.05) for fruits. The correlations between inattention and high consumption of dairy, meat, and high‐carbohydrate food were even weaker, ranging from 0.001 to 0.02. Hyperactivity/impulsivity and dietary habits showed similar correlation patterns to inattention, but the magnitude was smaller, with positive correlations ranging between 0.03 (95% CI: 0.01, 0.05) for high‐fat food and 0.09 (95% CI: 0.06, 0.11) for high‐sugar food, and negative correlations ranging from −0.02 (95% CI: −0.04, −0.01) for vegetables to −0.03 (95% CI: −0.05, −0.01) for fruits. For both inattention and hyperactivity/impulsivity, the strongest correlations with dietary habits were consistently observed for high‐sugar food and unhealthy food.

**TABLE 1:** Means and standard deviations (*SD*) for ADHD scales and total frequency^a^ of various diets, by age, sex and SES group

	Age			Sex		SES	
	20–29 (n = 5,969)	30–39 (n = 6,781)	40–47 (n = 5,249)	Male (n = 7,216)	Female (n = 10,783)	Low (n = 3,146)	High (n = 10,269)
IA	2.16 ± 2.15	1.90 ± 2.05	1.84 ± 2.04	2.02 ± 2.08	1.92 ± 2.09	2.22 ± 2.19	1.80 ± 1.99
HI	2.15 ± 2.11	1.98 ± 2.07	1.80 ± 2.02	1.98 ± 2.09	1.99 ± 2.06	2.10 ± 2.14	1.89 ± 2.02
Food groups	(n = 2,939–3,116)	(n = 3,299–3,370)	(n = 2,626–2,680)	(n = 3,297–3,352)	(n = 5,567–5,813)	(n = 1,697–1745)	(n = 4,969–5,112)
Fruits	1.39 ± 1.26	1.54 ± 1.32	1.61 ± 1.41	1.16 ± 1.08	1.71 ± 1.42	1.38 ± 1.32	1.59 ± 1.34
Vegetables	2.66 ± 1.98	3.08 ± 2.20	3.33 ± 2.24	2.57 ± 1.87	3.26 ± 2.27	2.74 ± 2.07	3.20 ± 2.16
Dairy	7.18 ± 4.41	7.03 ± 4.11	6.82 ± 4.19	7.66 ± 4.56	6.65 ± 3.99	7.42 ± 4.63	6.84 ± 4.01
Meat	1.37 ± 0.90	1.45 ± 0.83	1.44 ± 0.91	1.55 ± 1.00	1.35 ± 0.79	1.43 ± 0.84	1.43 ± 0.83
Seafood	0.47 ± 0.42	0.54 ± 0.46	0.59 ± 0.48	0.56 ± 0.48	0.52 ± 0.44	0.52 ± 0.46	0.54 ± 0.43
Rich in macro nutrients	(n = 3,107–3,117)	(n = 3,364–3,371)	(n = 2,672–2,685)	(n = 3,345–3,357)	(n = 5,798–5,815)	(n = 1739–1747)	(n = 5,102–5,114)
High in fat	2.70 ± 1.93	2.84 ± 1.83	3.01 ± 1.85	3.20 ± 1.98	2.63 ± 1.79	2.99 ± 2.01	2.82 ± 1.80
High in carbohydrates	10.87 ± 4.56	11.45 ± 4.60	11.94 ± 4.50	11.46 ± 4.67	11.36 ± 4.52	11.32 ± 4.85	11.51 ± 4.41
High in sugar	2.98 ± 2.28	2.72 ± 2.14	2.52 ± 2.08	3.22 ± 2.43	2.48 ± 1.97	3.05 ± 2.41	2.54 ± 1.99
High in protein	9.42 ± 4.94	9.35 ± 4.55	9.13 ± 4.58	10.20 ± 5.12	8.80 ± 4.35	9.73 ± 5.15	9.12 ± 4.41
Dietary patterns	(n = 3,108–3,110)	(n = 3,367–3,368)	(n = 2,674–2,679)	(n = 3,348–3,353)	(n = 2,801–5,804)	(n = 1740–1744)	(n = 5,103–5,106)
Unhealthy dietary pattern	3.92 ± 2.52	3.83 ± 2.27	3.66 ± 2.24*	4.37 ± 2.63	3.49 ± 2.11*	4.07 ± 2.56	3.66 ± 2.16*
Healthy dietary pattern	5.58 ± 3.36	6.33 ± 3.70	6.76 ± 3.70*	5.40 ± 3.14	6.67 ± 3.80*	5.72 ± 3.44	6.55 ± 3.64*

Abbreviations: HI, hyperactivity/impulsivity; IA, inattention.

^a^ Times or servings/day.

### 3.2 Sensitivity analysis

The correlation patterns between ADHD symptoms and different dietary habits were stable across age, sex, and SES. See Figures S2, S3, S4 and Table S4.

As is shown in Table 2, the correlations between inattention and different dietary habits were similar, after adjusting for hyperactivity/impulsivity in the partial pairwise Pearson's correlation models. The strongest correlations with inattention were still found in high‐sugar food (*r* = .10, 95%CI: 0.08, 0.12) and unhealthy food (*r* = .09, 95%CI: 0.07, 0.11). In contrast, the correlations between hyperactivity/impulsivity and different dietary habits were largely attenuated after controlling for inattention. The only statistically significant correlations with hyperactivity/impulsivity, after adjusting for inattention were found in in high‐sugar food (*r* = .03, 95% CI: 0.01, 0.06) and unhealthy food (*r* = .04, 95% CI: 0.01, 0.06), but the associations were very weak.

**TABLE 2:** The correlations with 95% confidence intervals between ADHD trait dimensions and different dietary habits, adjusted for relatedness of individuals and the other trait in each model, *N* = 8,731

		IA^a^	HI^b^
Food groups	Fruits	−0.06 (−0.08,‐0.04)	0.00 (−0.02,0.02)
	Vegetables	−0.06 (−0.08,‐0.04)	0.01 (−0.01,0.03)
	Dairy	0.01 (−0.01,0.03)	0.02 (−0.00,0.04)
	Meat	0.00 (−0.02,0.03)	0.01 (−0.01,0.03)
	Seafood	0.02 (−0.00,0.04)	0.02 (0.00,0.05)
Rich in macro nutrients	High in fat	0.05 (0.03,0.07)	0.01 (−0.02,0.03)
	High in carbohydrates	−0.00 (−0.03,0.02)	0.00 (−0.02,0.02)
	High in sugar	0.10 (0.08,0.12)	0.03 (0.01,0.06)
	High in protein	0.02 (−0.00,0.04)	0.03 (0.00,0.05)
Dietary patterns	Unhealthy dietary pattern	0.09 (0.07,0.11)	0.04 (0.01,0.06)
	Healthy dietary pattern	−0.06 (−0.08,‐0.04)	0.01 (−0.01,0.03)

Abbreviations: HI, Hyperactivity/impulsivity; IA, Inattention.

^a^ Adjusted hyperactivity‐impulsivity for the correlations between inattention and dietary habits.

^b^ Adjusted inattention for the correlations between hyperactivity‐impulsivity and dietary habits.

### 3.3 Genetically informative associations

Based on the phenotypic correlations, and to maximize statistical power, the twin analyses focused on ADHD symptoms dimensions, high‐sugar food and unhealthy dietary habits, and were collapsed across age, sex, and SES.

Twin correlations for each trait and CTCT correlations are shown in Table 3. We found higher CTCT correlations in MZ twins than in DZ twins, indicating genetic influences on the covariance between ADHD symptoms and unhealthy diets. Nonshared environmental influences were also evident because MZ CTCT correlations were smaller than the phenotypic correlations.

**TABLE 3:** Intraclass correlations and cross‐twin cross‐trait correlations with 95% confidence intervals for ADHD trait dimensions and dietary habits

Intraclass correlations				
	IA	HI	High‐sugar food	Unhealthy dietary pattern
MZ	0.38 (0.33, 0.40)	0.39 (0.33, 0.40)	0.42 (0.33, 0.45)	0.42 (0.33, 0.45)
DZ	0.11 (0.07, 0.15)	0.14 (0.10, 0.18)	0.16 (0.09, 0.22)	0.16 (0.09, 0.23)

Cross‐twin cross‐trait correlations				
	IA‐high‐sugar	IA‐unhealthy dietary pattern	HI‐high‐sugar	HI‐unhealthy dietary pattern
MZ	0.06 (0.00, 0.11)	0.05 (0.00, 0.11)	0.04 (−0.02, 0.09)	0.02(−0.04, 0.07)
DZ	0.00 (−0.05, 0.05)	0.03 (−0.02, 0.08)	0.02 (−0.03, 0.07)	−0.01(−0.06, 0.04)

Abbreviations: IA: Inattention, HI: Hyperactivity/impulsivity.

The univariate model‐fitting results are displayed in Table S5. Table S6 provides the bivariate model fitting results of ADHD symptom dimensions and dietary habits. The parameter estimates of the ADE models and AE models were presented in Table S7 and Table 4, respectively. The broad‐sense heritability (A + D) was 37% for inattention and hyperactivity/impulsivity. For dietary habits, the heritability was estimated as 38% (95% CI: 33, 44) for high consumption of high‐sugar food and 36% (95% CI: 31, 42) for unhealthy dietary habits. AE models provided the best fit to the data on ADHD symptom dimensions and dietary habits. Genetic correlations, except for hyperactivity/impulsivity and unhealthy food, were statistically significant, ranging from 0.09 (95% CI: 0.002, 0.19) for hyperactivity/impulsivity and high‐sugar food to 0.16 (95% CI: 0.07, 0.25) for inattention and high‐sugar food. All nonshared environmental correlations were statistically significant. The bivariate heritability estimates (the fraction of phenotypic covariance explained by genetic influences) were 44% (95% CI: 18, 70), 40% (95% CI: 10, 69), and 37% (95% CI: 1, 71) for inattention and high‐sugar food, inattention and unhealthy dietary pattern, and hyperactivity/impulsivity and high‐sugar food, respectively.

**TABLE 4:** Estimates of genetic and environmental effect (95% confidence intervals) from bivariate AE models

	A	E	*r*_A_	*r*_E_	*r*_P_	Bivariate A	Bivariate E
IA and high‐sugar food							
IA	0.34 (0.30, 0.37)	0.66 (0.63, 0.70)	.16 (.07, .25)	.11 (.06, .16)	.13 (.11, .15)	0.44 (0.18, 0.70)	0.56 (0.30, 0.82)
High‐sugar food	0.38 (0.33.0.43)	0.62 (0.56, 0.67)					
IA and unhealthy dietary pattern							
IA	0.34 (0.30, 0.37)	0.66 (0.63, 0.70)	.13 (.03, .22)	.10 (.05, .15)	.11 (.09, .13)	0.40 (0.10, 0.70)	0.60 (0.30, 0.90)
Unhealthy food	0.36 (0.31, 0.42)	0.64 (0.58, 0.69)					
HI and high‐sugar food							
HI	0.35 (0.32, 0.38)	0.65 (0.62, 0.68)	.09 (.002, .19)	.09 (.04, .15)	.09 (.07, .11)	0.37 (0.01, 0.71)	0.63 (0.30, 0.99)
High‐sugar food	0.38 (0.33, 0.44)	0.62 (0.56, 0.67)					
HI and unhealthy dietary pattern							
HI	0.35 (0.32, 0.38)	0.65 (0.62, 0.68)	.05 (−.05, .14)	.11 (.06, .16)	.09 (.07, .11)	0.20 (−0.21, 0.56)	0.80 (0.44, 1.20)
Unhealthy food	0.36 (0.31, 0.42)	0.64 (0.58, 0.69)					

*Note:* Bivariate A (bivariate heritability) refers to the amount of covariance between the two phenotypes explained by A, similarly for E.

Abbreviations: A, additive genetic factors; D, dominant genetic factors; E, nonshared environmental factors; HI, hyperactivity/impulsivity; IA, inattention.

In the MZ twin intrapair differences model (Table 5), correlations of the intrapair differences in ADHD symptoms and the intrapair differences in dietary habits, except for hyperactivity/impulsivity and high‐sugar food, were statistically significant, ranging from 0.08 (95% CI: 0.01, 0.15) to 0.13 (95% CI: 0.05, 0.20). Therefore, in a genetically identical twin pair, the twin who has more ADHD symptoms is likely to also consumes more high sugar and unhealthy food than his/her twin.

**TABLE 5:** Results from MZ Twin intrapair differences model

Differences (Twin1‐Twin2)		*N*	Correlation	95%ci	*p*‐value
IA	High‐sugar food	708	0.13	(0.05, 0.20)	<.01
IA	Unhealthy food	709	0.12	(0.04, 0,19)	<.01
HI	High‐sugar food	711	0.06	(−0.01, 0.14)	.09
HI	Unhealthy food	712	0.08	(0.01, 0.15)	.04

Abbreviations: HI, hyperactivity/impulsivity; IA, inattention.

## 4 DISCUSSION

In this nationwide population‐based sample of adult twins, we identified positive associations between self‐reported trait dimensions of ADHD and intake of seafood, high‐fat food, high‐sugar food, high‐protein food, and an unhealthy dietary pattern, and negative associations with consumption of fruits, vegetables, and a healthy dietary pattern. However, all of the associations are small in magnitude. These associations were stronger for inattention compared to hyperactivity/impulsivity. This pattern of associations was also reflected at the etiological level, where we found a slightly stronger genetic correlation of inattention with dietary habits than of hyperactivity/impulsivity with dietary habits. Nonshared environmental influences also contributed to the overlap between ADHD symptom dimensions and consumption of high‐sugar food and unhealthy dietary pattern. However, shared environmental influences probably contributed relatively little to the associations between ADHD symptoms and dietary habits. Our findings contribute to a better understanding of common etiological pathways between ADHD symptoms and various dietary habits.

At the phenotypic level, our results are in line with previous study from adults associating elevated levels of self‐reported ADHD symptoms with higher consumption of sweet food and lower consumption of vegetables and fruits (Weissenberger et al., 2018). However, the case–control study with 51 young adults (aged 18–25) suggested that nutrient intake was not associated with ADHD, but the conclusion was limited by the small sample size leading to low statistical power (Holton et al., 2019). Our results are also consistent with prior studies based on children and adolescents (Del‐Ponte, Quinte, Cruz, Grellert, & Santos, 2019), which may indicate that associations between ADHD symptoms and dietary habits are stable across the lifespan. We further found that all associations were consistent by age, sex, and SES. Given the chronic nature of ADHD related problems, people with ADHD are most likely exposed to unhealthy dietary factors across a substantial period of time, which may in part explain the well‐established increased risk for a variety of psychiatric and somatic morbidities (Weissenberger et al., 2017).

Our findings extend the previous literature in four important ways. First, the current study is the first to identify a dimension‐specific overlap between ADHD and different dietary habits, with a stronger correlation of inattention with dietary habits than hyperactivity/impulsivity with dietary habits. Support for a dimension specific association has also been observed for ADHD symptoms and binge‐eating behavior in adults (Capusan et al., 2017). One explanation for the minimal contribution of hyperactivity/impulsivity on dietary habits in adults is that hyperactive/impulsive symptoms tend to decrease at a higher rate with age compared with inattention symptoms (Willcutt et al., 2012). Future genomic studies on the association between ADHD and dietary habits may benefit from including information about ADHD symptom dimensions and/or subtypes.

Second, our results suggest the association between ADHD symptoms and different dietary habits is in part explained by shared genetic factors. Part of the genetic overlap may reflect genetic risk variants with general effects cutting across boundaries between neuropsychiatric traits and nutrition‐related or metabolic problems (Demontis et al., 2018; Meddens et al., 2018; Watson et al., 2019). Another more specific mechanism may involve the addictive potential of highly palatable foods (such as sweet, fatty, and salty foods). It is well established that the genetic liability of ADHD is in part shared with the genetic liability of several addiction disorders, such as alcoholism (Edwards & Kendler, 2012), pathological gambling (Comings et al., 1999), internet and videogame addiction (Weinstein & Weizman, 2012), and substance abuse (Zheng Chang, Lichtenstein, & Larsson, 2012). Our findings may therefore indicate that shared genetic factors of ADHD symptoms and dietary habits may partly reflect a common genetic pathway for different addictive behaviors. Typically, substance use disorders are explained by impulsivity (de Wit, 2009), although, in line with previous studies on other addictive behaviors (Burke, Loeber, White, Stouthamer‐Loeber, & Pardini, 2007; Wang, Yao, Zhou, Liu, & Lv, 2017) reported attention problems may potentially play a potent role more broadly in addictive behaviors beyond diet.

Third, nonshared environmental factors also contributed substantially to the overlap between ADHD symptoms and different dietary habits. Research has not previously focused on such factors and how they possibly contribute to the co‐occurrence of ADHD symptoms and different dietary habits. Some studies have reported that screen time and level of physical activity are associated with ADHD symptoms, and there is also support for a link with suggested dietary habits (Mian et al., 2019; Rios‐Hernandez, Alda, Farran‐Codina, Ferreira‐Garcia, & Izquierdo‐Pulido, 2017), but whether such factors explain the nonshared environmental overlap between ADHD symptom dimensions and dietary habits remains to investigated. Future research on nonshared environmental risk factors may not only aid in our understanding of the association between diet, ADHD and related lifestyle factors and disorders, but also help identify novel prevention and intervention target.

Fourth, although the associations between ADHD symptoms and dietary habits could be explained by common genetic or environmental determinants, the significant genetic and nonshared environmental correlations, and significant MZ twin intrapair differences also provided support for a potential causal link between inattention and dietary habits. However, due to the similarity of the etiology of the two traits, we were unable to further test the test the direction of causation in the current cross‐sectional twin data. Recently, a cohort study in Dutch children was the first to test bidirectional associations between ADHD symptoms and diets, indicating that children's ADHD symptoms predicted poor diet in later life, but that diet quality was not an independent predictor of later ADHD symptoms (Mian et al., 2019). Therefore, future longitudinal studies (e.g., cross‐lagged models) and various study designs (e.g., Mendelian randomization) are needed to replicate this finding in children and adolescents, and to explore the directions of effect in adults.

### 4.1 Strengths and limitations

Our study has several strengths, including but not limited to, a very large adult twin sample, focus on two separate ADHD trait dimensions and extensive dietary phenotyping as well as an exploration of potential modifying effects of age, sex, and SES.

Several limitations should be considered. First, the associations between ADHD symptoms and dietary habits might be biased toward null as it is known that self‐report among adults underestimates ADHD‐symptoms compared to reports from other informants (Brikell, Kuja‐Halkola, & Larsson, 2015; Merwood et al., 2013). Consequently, the heritability estimates for self‐reported ADHD symptoms in adults were consistent with prior research using self‐ratings (S. V. Faraone & Larsson, 2019; Larsson et al., 2013), but substantially lower than those based on multiple raters or a clinical ADHD diagnosis (Brikell et al., 2015; Z. Chang, Lichtenstein, Asherson, & Larsson, 2013; Larsson et al., 2014). Future research needs to resolve if observed phenotypic and genetic associations are stronger using other assessments of ADHD and dietary habits (e.g., 24‐hr calls, food records, or macronutrients assessment). Second, the response rate in our population‐based twin sample was moderate (59.6%), and FFQ as a voluntary section had a lower response rate of 36.8%. A previous study from our team (Larsson et al., 2013) explored differences between participants and nonparticipants in the STAGE sample, and revealed that females with higher SES and lower levels of behavioral problems were more likely to participate in the STAGE questionnaires. In contrast, when comparing STAGE participants that responded to both the ADHD questionnaire and the FFQ with those that only responded to the ADHD questionnaire, we found that females with lower SES and higher levels of behavior problems were more likely to respond to both. Thus, our phenotypic results may not generalize to the general population. However, a similar response rate was found in DZ and MZ twins, which indicates that bias due to nonrandom missing probably had little impact on the estimates of the relative contribution of genetic and environmental influences.

Overall, we have found evidence for both phenotypic and genetic correlations between ADHD symptoms and unhealthy dietary habits in adulthood. Future longitudinal studies with various assessments of ADHD and dietary habits are needed to explore the casual associations between ADHD symptoms and dietary habits in adults.

## CONFLICT OF INTEREST

Dr. Larsson has served as a speaker for Evolan and Shire and has received research grants from Shire; all outside the submitted work. The remaining authors declare that they have no conflict of interest.

## AUTHOR CONTRIBUTIONS

Lin Li, Katarina Bälter, and Henrik Larsson were responsible for the study concept and design. Lin Li performed the analyses under the supervision of Mark J. Taylor. Tor‐Arne Hegvik contributed to all the visualizations. Lin Li drafted the manuscript. Ralf Kuja‐Halkola, Qi Chen, Tor‐Arne Hegvik, Ashley E. Tate, Zheng Chang, Alejandro Arias‐Vásquez and Catharina A Hartman gave inputs regarding the overall interpretation of the method and results and provided critical revision of the manuscript. All authors critically reviewed content and approved the final version for publication.

## DATA AVAILABILITY STATEMENT

Research data are not shared.

## ETHICS STATEMENT

The authors assert that all procedures contributing to this work comply with the ethical standards of the relevant national and institutional committees on human experimentation and with the Helsinki Declaration of 1975, as revised in 2008.

## Supporting information


**Table S1** Supporting information

**Figure S1** Flow diagram for study participants

**Figure S2** Age‐specific associations (correlations with 95% confidence intervals) between ADHD trait dimensions and different dietary habits investigated in the primary analyses

**Figure S3** Sex‐specific associations (correlations with 95% confidence intervals) between ADHD trait dimensions and different dietary habits investigated in the primary analyses

**Figure S4** SES‐specific associations (correlations with 95% confidence intervals) between ADHD trait dimensions and different dietary habits investigated in the primary analyses
