# Supplementary material for: Attention‐deficit/hyperactivity disorder symptoms and dietary habits in adulthood: A large population‐based twin study in Sweden
Source: Am J Med Genet B Neuropsychiatr Genet. 2020 Oct 7;183(8):475–85. doi: 10.1002/ajmg.b.32825 (PMC7702140; doi:10.1002/ajmg.b.32825)
Supplement: Supplementary file 1 — Table S1 Supporting information [file AJMG-183-475-s001.docx]

**Table S1** Comparison between those with or without nutrition information

|  | Without nutrition information  (n=8,839) | With nutrition information  (n=9,160) | P |
| --- | --- | --- | --- |
| Age | 33.81±7.58 | 33.73±7.71 | 0.12* |
| Sex |  |  | 0.00# |
| Male | 3861 (53.51%) | 4978 (46.17%) |  |
| Female | 3355 (46.49%) | 5805 (53.83%) |  |
| SES |  |  | 0.00# |
| Low | 1401 (44.53%) | 5161 (50.26%) |  |
| High | 1745 (55.47%) | 5108 (49.74%) |  |
| ADHD |  |  |  |
| Inattention | 1.80±2.03 | 2.11±2.13 | 0.00* |
| Hyperactivity | 1.83±2.00 | 2.13±2.12 | 0.00* |
| Total score | 3.63±3.56 | 4.24±4.17 | 0.00* |
| Zygosity |  |  |  |
| MZ | 3381 (49.31%) | 5239 (48.74%) | 0.46 |
| DZ | 3457 (50.69%) | 5509 (51.26%) |  |

*P-value from Linear mixed effect model

# P-value from chi-square test.

**Table S2.** The definitions of dietary habits: 1) consumption of food groups, 2) consumption of food items rich in a particular macronutrient, and 3) healthy dietary patterns and unhealthy dietary patterns. The intake frequency for each food item was converted into number of servings per day and summarized for each food group, respectively.

| **Dietary habits** | **Food Groups** | **Food items** |
| --- | --- | --- |
| 1. Food Groups | Fruits | Orange, apple, banana, berries, other fruit |
|  | Vegetables | Salad, cabbage, cauliflower, broccoli, tomato, pepper, spinach, green peas, onion, garlic, mixed vegetables, carrot |
|  | Dairy | Cheese, low-fat cheese, cottage cheese, low-fat milk, milk 1.5% fat, regular milk, low-fat yoghurt, yoghurt |
|  | Meat | Minced meat/meatball, pork, beef, chicken, sausage, liver, liver pâté s, ham, lamb, other meat |
|  | Seafood | Herring/mackerel, salmon, fish fingers, tuna, other fish, caviar, prawns |
| 2.Food items ich in macronutrients | High in fat | Pizza, fried potato, French fries, chips, nuts, dressing, mayonnaise, cream, cheese |
|  | High in carbohydrates | bread (white bread, loaf of bread, wholemeal bread, crisp bread), oats porridge, porridge, cereal (sweet cereal, cereal, muesli), pasta, rice, wheat, boiled potato, pancakes, vegetables and fruits |
|  | High in sugar | Cookies, crackers, cake, chocolate, candy, ice cream, jam, berry cream, juice, energy drink, honey, ketchup |
|  | HIgh in protein | Minced meat/meatball, pork, beef, chicken, sausage, liver, liver pâté, ham, lamb, other meat, beans/lentils, tofu/soy, quorn, cheese, low-fat cheese, cottage cheese, low-fat milk, milk 1.5% fat, regular milk, low-fat yoghurt, yoghurt, Herring/mackerel, salmon, fish fingers, tuna, other fish, caviar, prawns, egg, high calorie drink/ protein drink |
| 3. Dietary patterns | Unhealthy dietary pattern | Pizza, sausage, fried potato, French fries, minced meat/meatball, pork, beef, Cookies, crackers, cake, chocolate, candy, ice cream, jam, berry cream, juice, energy drink, Hamburg, chips, dressing, mayonnaise, cream, ketchup |
|  | Healthy dietary pattern | Herring/mackerel, salmon, fish fingers, tuna, other fish, prawns, orange, apple, banana, berries, other fruit, salad, cabbage, cauliflower, broccoli, tomato, pepper, spinach, green peas, onion, garlic, mixed vegetables, carrot, beans/lentils, nuts, boiled potato, chicken, flax, sweet cereal, cereal, muesli |

***Table S3*** *The distributions of ADHD symptoms and dietary habits before and after log-transformation*

|  | N | Mean | Std Dev | Median | 25th Pctl | 75th Pctl | Skewness |
| --- | --- | --- | --- | --- | --- | --- | --- |
| *Raw variables* | | | | | | | |
| **ADHD symptoms** |  |  |  |  |  |  |  |
| Inattention | 17861 | 1.96 | 2.08 | 1.00 | 0.00 | 3.00 | 1.14 |
| Hyperactivity | 17866 | 1.98 | 2.07 | 1.00 | 0.00 | 3.00 | 1.06 |
| **Food groups** |  |  |  |  |  |  |  |
| Fruits | 9124 | 1.51 | 1.33 | 1.10 | 0.61 | 2.07 | 2.33 |
| Vegetables | 9142 | 3.01 | 2.16 | 2.52 | 1.55 | 3.94 | 2.41 |
| Dairy | 9165 | 7.02 | 4.24 | 6.20 | 4.20 | 9.20 | 1.30 |
| Meat | 8864 | 1.42 | 0.88 | 1.27 | 0.94 | 1.72 | 4.56 |
| Seafood | 9120 | 0.53 | 0.46 | 0.42 | 0.28 | 0.67 | 4.84 |
| **Rich in macro nutrients** |  |  |  |  |  |  |  |
| High in fat | 9164 | 2.84 | 1.88 | 2.62 | 2.00 | 3.28 | 0.83 |
| High in carbohydrates | 9171 | 11.40 | 4.58 | 10.80 | 8.37 | 13.65 | 1.42 |
| High in sugar | 9143 | 2.75 | 2.18 | 2.24 | 1.32 | 3.55 | 2.11 |
| High in protein | 9168 | 9.31 | 4.70 | 8.50 | 6.05 | 11.69 | 1.42 |
| **Dietary patterns** |  |  |  |  |  |  |  |
| Unhealthy dietary pattern | 9149 | 3.81 | 2.35 | 3.29 | 2.31 | 4.63 | 2.33 |
| Healthy dietary pattern | 9157 | 6.20 | 3.62 | 5.51 | 3.75 | 7.91 | 2.19 |
|  |  |  |  |  |  |  |  |
| *Log-transformed variables* | | | | | | | |
| **ADHD symptoms** |  |  |  |  |  |  |  |
| Inattention | 17861 | 0.84 | 0.70 | 0.69 | 0.00 | 1.39 | 0.14 |
| Hyperactivity | 17866 | 0.85 | 0.70 | 0.69 | 0.00 | 1.39 | 0.11 |
| **Food groups** |  |  |  |  |  |  |  |
| Fruits | 9124 | 0.81 | 0.44 | 0.74 | 0.48 | 1.12 | 0.60 |
| Vegetables | 9142 | 1.27 | 0.48 | 1.26 | 0.94 | 1.60 | 0.19 |
| Dairy | 9165 | 1.94 | 0.57 | 1.97 | 1.65 | 2.32 | -0.70 |
| Meat | 8864 | 0.84 | 0.30 | 0.82 | 0.66 | 1.00 | 0.64 |
| Seafood | 9120 | 0.39 | 0.23 | 0.35 | 0.25 | 0.51 | 1.41 |
| **Rich in macro nutrients** |  |  |  |  |  |  |  |
| High in fat | 9164 | 1.22 | 0.52 | 1.29 | 1.10 | 1.45 | -0.40 |
| High in carbohydrates | 9171 | 2.45 | 0.37 | 2.47 | 2.24 | 2.68 | -0.52 |
| High in sugar | 9143 | 1.19 | 0.51 | 1.18 | 0.84 | 1.52 | 0.29 |
| High in protein | 9168 | 2.24 | 0.45 | 2.25 | 1.95 | 2.54 | -0.33 |
| **Dietary patterns** |  |  |  |  |  |  |  |
| Unhealthy dietary pattern | 9149 | 1.48 | 0.42 | 1.46 | 1.20 | 1.73 | 0.31 |
| Healthy dietary pattern | 9157 | 1.86 | 0.47 | 1.87 | 1.56 | 2.19 | -0.11 |

**Table S4**. The correlations with 95% confidence intervals between ADHD symptoms and different dietary habits (adjusted the relatedness of individuals), stratified by age, sex and SES

|  |  | IA | HI |
| --- | --- | --- | --- |
| *Stratified by age* |  | *20-29 years of age (N=2901)* |  |
| 1)Food groups | Fruits | -0.10 (-0.14,-0.07) | -0.03 (-0.06,0.01) |
|  | Vegetables | -0.04 (-0.08,-0.01) | -0.00 (-0.04,0.03) |
|  | Dairy | 0.01 (-0.02,0.05) | 0.01 (-0.02,0.05) |
|  | Meat | 0.02 (-0.02,0.06) | 0.02 (-0.02,0.05) |
|  | Seafood | 0.03 (-0.01,0.07) | 0.01 (-0.02,0.05) |
| 2)Food items rich in macro nutrients | High in fat | 0.08 (0.04,0.11) | 0.03 (-0.01,0.07) |
|  | High in carbohydrates | 0.01 (-0.02,0.05) | 0.01 (-0.03,0.04) |
|  | High in sugar | 0.13 (0.09,0.16) | 0.09 (0.05,0.13) |
|  | High in protein | 0.03 (-0.01,0.06) | 0.03 (-0.01,0.06) |
| 3)Dietary patterns | Unhealthy dietary pattern | 0.12 (0.09,0.16) | 0.10 (0.06,0.13) |
|  | Healthy dietary pattern | -0.06 (-0.10,-0.03) | -0.01 (-0.05,0.02) |
|  |  | *30-39 years of age (N=3254)* |  |
| 1)Food groups | Fruits | -0.05 (-0.08,-0.01) | -0.01 (-0.05,0.02) |
|  | Vegetables | -0.05 (-0.08,-0.01) | -0.00 (-0.04,0.03) |
|  | Dairy | 0.02 (-0.02,0.05) | 0.04 (0.01,0.08) |
|  | Meat | 0.02 (-0.02,0.05) | 0.01 (-0.02,0.05) |
|  | Seafood | 0.04 (0.01,0.08) | 0.05 (0.01,0.08) |
| 2)Food items rich in macro nutrients | High in fat | 0.03 (-0.01,0.06) | 0.04 (0.01,0.08) |
|  | High in carbohydrates | -0.00 (-0.04,0.03) | 0.01 (-0.02,0.05) |
|  | High in sugar | 0.13 (0.09,0.16) | 0.08 (0.04,0.11) |
|  | High in protein | 0.03 (-0.01,0.06) | 0.05 (0.02,0.08) |
| 3)Dietary patterns | Unhealthy dietary pattern | 0.11 (0.08,0.14) | 0.06 (0.03,0.10) |
|  | Healthy dietary pattern | -0.04 (-0.08,-0.01) | -0.00 (-0.04,0.03) |
|  |  | *40-47 years of age (N=2576)* |  |
| 1)Food groups | Fruits | -0.05 (-0.09,-0.01) | -0.03 (-0.07,0.01) |
|  | Vegetables | -0.07 (-0.11,-0.03) | -0.02 (-0.06,0.01) |
|  | Dairy | 0.04 (0.00,0.08) | 0.02 (-0.02,0.06) |
|  | Meat | -0.01 (-0.05,0.03) | 0.01 (-0.03,0.04) |
|  | Seafood | 0.02 (-0.02,0.06) | 0.06 (0.02,0.10) |
| 2)Food items rich in macro nutrients | High in fat | 0.07 (0.04,0.11) | 0.02 (-0.02,0.06) |
|  | High in carbohydrates | -0.02 (-0.06,0.02) | -0.01 (-0.05,0.02) |
|  | High in sugar | 0.12 (0.08,0.16) | 0.08 (0.04,0.12) |
|  | High in protein | 0.05 (0.01,0.08) | 0.03 (-0.01,0.07) |
| 3)Dietary patterns | Unhealthy dietary pattern | 0.10 (0.06,0.13) | 0.07 (0.04,0.11) |
|  | Healthy dietary pattern | -0.06 (-0.10,-0.02) | -0.02 (-0.06,0.02) |
| *Stratified by sex* |  | *Male (N=3247)* |  |
| 1)Food groups | Fruits | -0.07 (-0.11,-0.04) | -0.05 (-0.08,-0.01) |
|  | Vegetables | -0.06 (-0.10,-0.03) | -0.03 (-0.06,0.00) |
|  | Dairy | 0.04 (0.00,0.07) | 0.03 (-0.00,0.07) |
|  | Meat | 0.00 (-0.03,0.04) | 0.04 (0.01,0.08) |
|  | Seafood | 0.00 (-0.03,0.04) | 0.03 (-0.01,0.06) |
| 2)Food items rich in macro nutrients | High in fat | 0.06 (0.03,0.09) | 0.03 (-0.01,0.06) |
|  | High in carbohydrates | -0.02 (-0.06,0.01) | -0.02 (-0.05,0.02) |
|  | High in sugar | 0.09 (0.06,0.13) | 0.10 (0.06,0.13) |
|  | High in protein | 0.04 (0.00,0.07) | 0.05 (0.02,0.08) |
| 3)Dietary patterns | Unhealthy dietary pattern | 0.08 (0.05,0.11) | 0.10 (0.06,0.13) |
|  | Healthy dietary pattern | -0.07 (-0.10,-0.03) | -0.04 (-0.07,-0.00) |
|  |  | *Female (N=5484)* |  |
| 1)Food groups | Fruits | -0.06 (-0.08,-0.03) | -0.02 (-0.04,0.01) |
|  | Vegetables | -0.05 (-0.07,-0.02) | -0.01 (-0.03,0.02) |
|  | Dairy | 0.01 (-0.02,0.03) | 0.02 (-0.00,0.05) |
|  | Meat | 0.00 (-0.02,0.03) | -0.01 (-0.04,0.02) |
|  | Seafood | 0.04 (0.02,0.07) | 0.04 (0.01,0.07) |
| 2)Food items rich in macro nutrients | High in fat | 0.04 (0.02,0.07) | 0.03 (0.00,0.06) |
|  | High in carbohydrates | 0.01 (-0.02,0.03) | 0.01 (-0.02,0.03) |
|  | High in sugar | 0.14 (0.11,0.17) | 0.08 (0.05,0.10) |
|  | High in protein | 0.02 (-0.01,0.05) | 0.03 (0.00,0.06) |
| 3)Dietary patterns | Unhealthy dietary pattern | 0.12 (0.09,0.15) | 0.07 (0.04,0.10) |
|  | Healthy dietary pattern | -0.05 (-0.07,-0.02) | -0.01 (-0.03,0.02) |
| *Stratified by SES* |  | *Low SES (N=1660)* |  |
| 1)Food groups | Fruits | -0.08 (-0.13,-0.04) | -0.07 (-0.12,-0.02) |
|  | Vegetables | -0.08 (-0.12,-0.03) | -0.04 (-0.08,0.01) |
|  | Dairy | 0.02 (-0.02,0.07) | 0.03 (-0.01,0.08) |
|  | Meat | 0.03 (-0.02,0.08) | 0.01 (-0.04,0.05) |
|  | Seafood | 0.02 (-0.03,0.07) | 0.03 (-0.01,0.08) |
| 2)Food items rich in macro nutrients | High in fat | 0.03 (-0.02,0.08) | 0.04 (-0.01,0.09) |
|  | High in carbohydrates | -0.02 (-0.07,0.03) | -0.02 (-0.07,0.03) |
|  | High in sugar | 0.15 (0.11,0.20) | 0.11 (0.06,0.16) |
|  | High in protein | 0.04 (-0.01,0.08) | 0.04 (-0.01,0.09) |
| 3)Dietary patterns | Unhealthy dietary pattern | 0.13 (0.08,0.18) | 0.11 (0.06,0.15) |
|  | Healthy dietary pattern | -0.08 (-0.13,-0.03) | -0.05 (-0.10,-0.00) |
|  |  | *High SES (N=4917)* |  |
| 1)Food groups | Fruits | -0.04 (-0.07,-0.02) | -0.02 (-0.04,0.01) |
|  | Vegetables | -0.04 (-0.07,-0.02) | -0.01 (-0.04,0.02) |
|  | Dairy | 0.01 (-0.01,0.04) | 0.03 (-0.00,0.06) |
|  | Meat | -0.00 (-0.03,0.03) | 0.00 (-0.02,0.03) |
|  | Seafood | 0.03 (0.00,0.06) | 0.05 (0.02,0.07) |
| 2)Food items rich in macro nutrients | High in fat | 0.06 (0.03,0.09) | 0.03 (0.00,0.06) |
|  | High in carbohydrates | -0.01 (-0.03,0.02) | -0.00 (-0.03,0.02) |
|  | High in sugar | 0.11 (0.08,0.14) | 0.07 (0.04,0.10) |
|  | High in protein | 0.02 (-0.00,0.05) | 0.04 (0.01,0.07) |
| 3)Dietary patterns | Unhealthy dietary pattern | 0.10 (0.07,0.12) | 0.06 (0.04,0.09) |
|  | Healthy dietary pattern | -0.04 (-0.07,-0.01) | -0.00 (-0.03,0.02) |

IA: Inattention, HI: Hyperactivity-impulsivity

**Table S5**. Model-fitting results of univariate analysis of ADHD symptoms and dietary habits

|  |  |  | | Fit of model compared to saturated model | | | | | |
| --- | --- | --- | --- | --- | --- | --- | --- | --- | --- |
|  |  | -2LL | df | | Compare to | ꭓ^2^ | ∆df | *P*-value | AIC |
| IA | Saturated model | 44674.73 | 17461 | |  |  |  |  | 9752.73 |
|  | ACE model | 44690.27 | 17467 | | Saturated | 15.54 | 6 | 0.016 | 9756.27 |
|  | **ADE model** | **44677.65** | **17467** | | **Saturated** | **2.92** | **6** | **0.819** | **9743.65** |
|  | AE model | 44690.27 | 17468 | | ADE | 12.62 | 1 | 0.003 | 9754.27 |
|  | E model | 45050.41 | 17469 | | ADE | 372.76 | 2 | <0.001 | 10112.41 |
| HI | Saturated model | 44603.06 | 17468 | |  |  |  |  | 9667.06 |
|  | ACE model | 44611.30 | 17474 | | Saturated | 8.24 | 6 | 0.221 | 9663.30 |
|  | **ADE model** | **44605.99** | **17474** | | **Saturated** | **2.93** | **6** | **0.817** | **9657.99** |
|  | AE model | 44611.30 | 17475 | | ADE | 5.31 | 1 | 0.021 | 9661.30 |
|  | E model | 45005.83 | 17476 | | ADE | 399.84 | 2 | <0.001 | 10053.83 |
| High-sugar food | Saturated model | 15974.89 | 8916 | |  |  |  |  | -1857.11 |
|  | ACE model | 15979.53 | 8922 | | Saturated | 4.64 | 6 | 0.591 | -1864.47 |
|  | ADE model | 15978.33 | 8922 | | Saturated | 3.44 | 6 | 0.752 | -1865.67 |
|  | **AE model** | **15979.53** | **8923** | | **ADE** | **1.20** | **1** | **0.273** | **-1866.47** |
|  | E model | 16121.66 | 8924 | | ADE | 143.33 | 2 | <0.001 | -1726.34 |
| Unhealthy food | Saturated model | 14150.54 | 8922 | |  |  |  |  | -3693.46 |
|  | ACE model | 14153.12 | 8928 | | Saturated | 2.58 | 6 | 0.860 | -3702.88 |
|  | ADE model | 14152.74 | 8928 | | Saturated | 2.20 | 6 | 0.900 | -3703.26 |
|  | **AE model** | **14153.12** | **8929** | | **ADE** | **0.38** | **1** | **0.532** | **-3704.88** |
|  | E model | 14290.40 | 8930 | | ADE | 137.66 | 2 | <0.001 | -3569.60 |

IA: Inattention, HI: Hyperactivity-impulsivity, LL: Log Likelihood; df: degree of freedom; AIC: Akaike’s Information Criterion. Best-fitting models indicated in bold.

**Table S6**. Model-fitting results of bivariate analysis of ADHD symptoms and dietary habits

|  |  | Fit of model compared to saturated model | | | | | |  |
| --- | --- | --- | --- | --- | --- | --- | --- | --- |
|  |  | -2LL | df | Compare to | ꭓ^2^ | ∆df | *P*-value | AIC |
| IA and High-sugar food | | | | | | | |  |
|  | Saturated model | 60503.66 | 26369 |  |  |  |  | 7765.66 |
|  | ACE model | 60529.73 | 26386 | Saturated | 26.07 | 17 | 0.073 | 7757.73 |
|  | **ADE model** | **60515.51** | **26386** | **Saturated** | **11.84** | **17** | **0.810** | **7743.51** |
|  | AE model | 60529.73 | 26389 | ADE | 14.23 | 3 | 0.003 | 7751.73 |
|  | E model | 61029.22 | 26392 | ADE | 513.71 | 6 | <0.001 | 8245.22 |
| IA and Unhealthy dietary pattern | |  |  |  |  |  |  |  |
|  | Saturated model | 58717.53 | 26375 |  |  |  |  | 5967.53 |
|  | ACE model | 58738.28 | 26392 | Saturated | 20.75 | 17 | 0.238 | 5954.28 |
|  | **ADE model** | **58724.75** | **26392** | **Saturated** | **7.22** | **17** | **0.981** | **5940.74** |
|  | AE model | 58738.28 | 26395 | ADE | 13.53 | 3 | 0.004 | 5948.28 |
|  | E model | 59233.25 | 26398 | ADE | 508.50 | 6 | <0.001 | 6437.25 |
| HI and High-sugar food | |  |  |  |  |  |  |  |
|  | Saturated model | 60496.57 | 26376 |  |  |  |  | 7744.57 |
|  | ACE model | 60512.86 | 26393 | Saturated | 16.29 | 17 | 0.503 | 7726.86 |
|  | ADE model | 60506.28 | 26393 | Saturated | 9.71 | 17 | 0.915 | 7720.28 |
|  | **AE model** | **60512.86** | **26396** | **ADE** | **6.58** | **3** | **0.087** | **7720.86** |
|  | E model | 61049.52 | 26399 | ADE | 543.25 | 6 | <0.001 | 8251.52 |
| HI and Unhealthy dietary pattern | |  |  |  |  |  |  |  |
|  | Saturated model | 27970.57 | 26382 |  |  |  |  | -24793.43 |
|  | ACE model | 27981.90 | 26399 | Saturated | 11.33 | 17 | 0.839 | -24816.11 |
|  | ADE model | 27976.55 | 26399 | Saturated | 5.98 | 17 | 0.9939 | -24821.45 |
|  | **AE model** | 27981.90 | 26402 | **ADE** | 5.35 | 3 | 0.149 | -24822.11 |
|  | E model | 28424.32 | 26405 | ADE | 447.77 | 6 | <0.001 | 24385.68 |

IA: Inattention, HI: Hyperactivity-impulsivity, LL: Log Likelihood; df: degree of freedom; AIC: Akaike’s Information Criterion. Best-fitting models indicated in bold.

**Table S7**. Estimates of genetic and environmental effect (95% confidence intervals) from bivariate ADE models.

|  |  | A | D | A+D | E | r_A_ | r_D_ | r_A+D_ | r_E_ | r_P_ | Bivariate A | Bivariate D | Bivariate A+D | Bivariate E |
| --- | --- | --- | --- | --- | --- | --- | --- | --- | --- | --- | --- | --- | --- | --- |
| ***ADE model*** | |  |  |  |  |  |  |  |  |  |  |  |  |  |
| IA and High-sugar food | | |  |  |  |  |  |  |  |  |  |  |  |  |
|  | IA | 0.08  (0,0.22) | 0.29  (0.13,0.39) | 0.36  (0.33,0.40) | 0.64  (0.60, 0.67) | 0.37  (-1,1) | 0.04  (-1,1) | 0.15  (0.05,0.24) | 0.11  (0.06,0.17) | 0.13  (0.11,0.15) | 0.40  (-0.82,1.54) | 0.06  (-1.20,1.36) | 0.45  (0.16,0.72) | 0.55  (0.28,0.84) |
|  | High-sugar food | 0.23  (0,0.43) | 0.17  (0,0.43) | 0.40  (0.34,0.45) | 0.60  (0.55,0,66) |  |  |  |  |  |  |  |  |  |
| IA and Unhealthy dietary pattern | | |  |  |  |  |  |  |  |  |  |  |  |  |
|  | IA | 0.08  (0,0.22) | 0.29  (0.13,0.39) | 0.36  (0.33,0.40) | 0.64  (0.60, 0.67) | -0.17  (-1,1) | 0.41  (-1,1) | 0.14  (0.04,0.24) | 0.09  (-0.04,0.15) | 0.11  (0.09,0.13) | -0.22  (-1.51,1.15) | 0.69  (-0.08,2.08) | 0.47  (0.14,0.79) | 0.53  (0.21,0.86) |
|  | Unhealthy food | 0.26  (0,0.41) | 0.12  (0,0.40) | 0.37  (0.31,0.43) | 0.63  (0.57,0.68) |  |  |  |  |  |  |  |  |  |
|  |  |  |  |  |  |  |  |  |  |  |  |  |  |  |
| HI and High-sugar food | | |  |  |  |  |  |  |  |  |  |  |  |  |
|  | HI | 0.19  (0.04,0.33) | 0.18  (0.03,0.34) | 0.37  (0.33,0.40) | 0.63  (0.60, 0.67) | 0.003  (-1,1) | 0.22  (-1,1) | 0.09  (0.03,0.15) | 0.09  (0.03,0.15) | 0.09  (0.07,0.11) | 0.004  (-1,1) | 0.40  (-1,1) | 0.40  (0.01,0.70) | 0.60  (0.22,0.99) |
|  | High-sugar food | 0.23  (0,0.43) | 0.17  (0,0.44) | 0.40  (0.34,0.45) | 0.60  (0.55,0.66) |  |  |  |  |  |  |  |  |  |
| HI and Unhealthy dietary pattern | | |  |  |  |  |  |  |  |  |  |  |  |  |
|  | HI | 0.19  (0.04,0.33) | 0.18  (0.03,0.34) | 0.37  (0.33,0.40) | 0.63  (0.60, 0.67) | -0.38  (-1,0.28) | 0.77  (-1,1) | 0.08  (-0.02,0.17) | 0.09  (0.04,0.15) | 0.09  (0.07,0.11) | -0.94  (-1,1) | 1.26  (-1,2.83) | 0.32  (-0.02,0.17) | 0.68  (0.29,1.10) |
|  | Unhealthy food | 0.26  (0.01,0.40) | 0.11  (0,0.38) | 0.37  (0.31,0.43) | 0.63  (0.57,0.69) |  |  |  |  |  |  |  |  |  |

A: additive genetic factors, D: dominant genetic factors, E: non-shared environmental factors, IA: Inattention, HI: Hyperactivity-impulsivity,

Bivariate A (bivariate heritability) refers to the amount of covariance between the two phenotypes explained by A, similarly for E.
