# Supplementary figures and images for: Attention‐deficit/hyperactivity disorder symptoms and dietary habits in adulthood: A large population‐based twin study in Sweden
Source: Am J Med Genet B Neuropsychiatr Genet. 2020 Oct 7;183(8):475–85. doi: 10.1002/ajmg.b.32825 (PMC7702140; doi:10.1002/ajmg.b.32825)

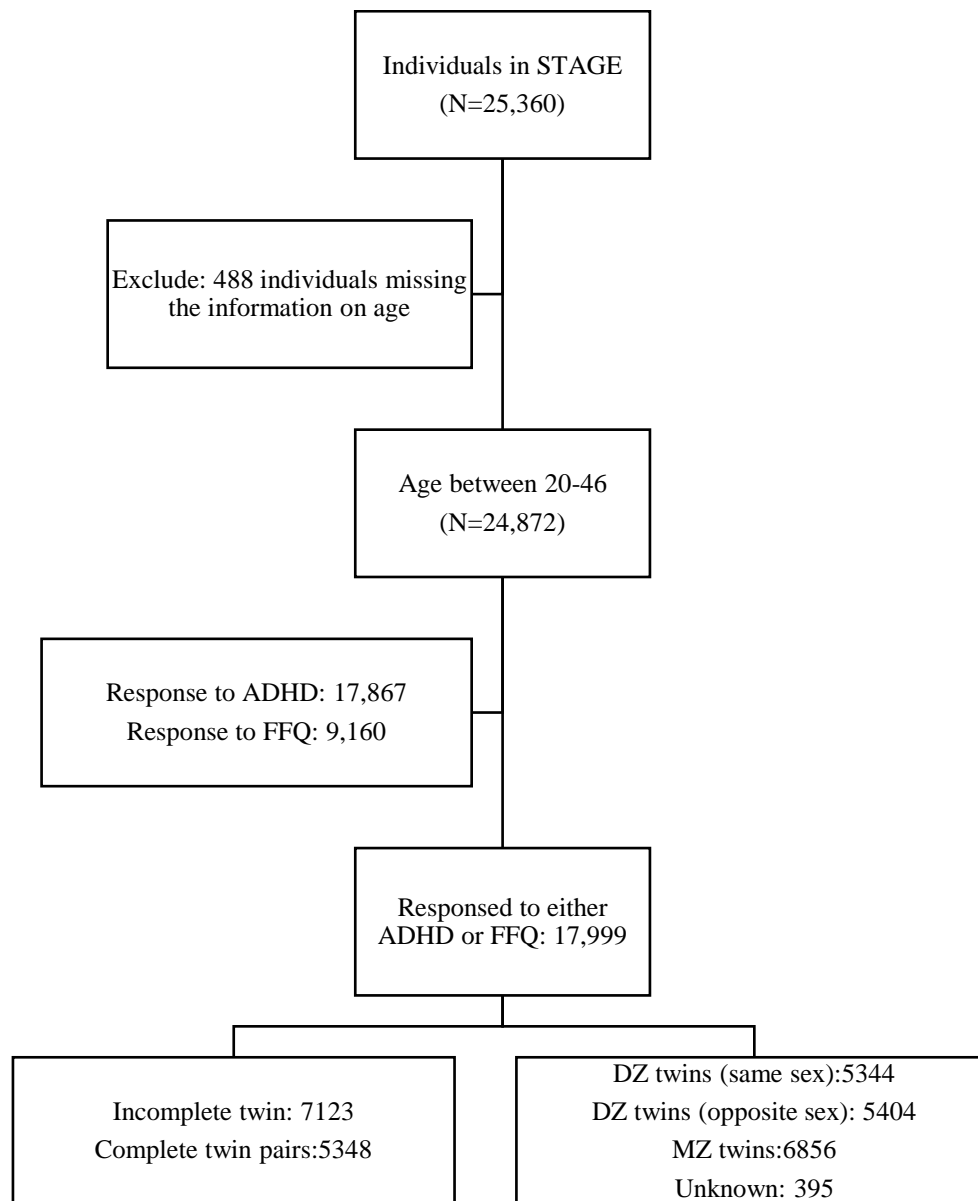

**Figure S1.** Flow diagram for study participants

Supplement: Supplementary file 2 — Figure S1 Flow diagram for study participants [file AJMG-183-475-s002.pdf]
