# Supplementary material for: Attention‐deficit/hyperactivity disorder symptoms and dietary habits in adulthood: A large population‐based twin study in Sweden
Source: Am J Med Genet B Neuropsychiatr Genet. 2020 Oct 7;183(8):475–85. doi: 10.1002/ajmg.b.32825 (PMC7702140; doi:10.1002/ajmg.b.32825)
Supplement: Supplementary file 5 — Figure S4 SES‐specific associations (correlations with 95% confidence intervals) between ADHD trait dimensions and different dietary habits investigated in the primary analyses [file AJMG-183-475-s005.pdf]

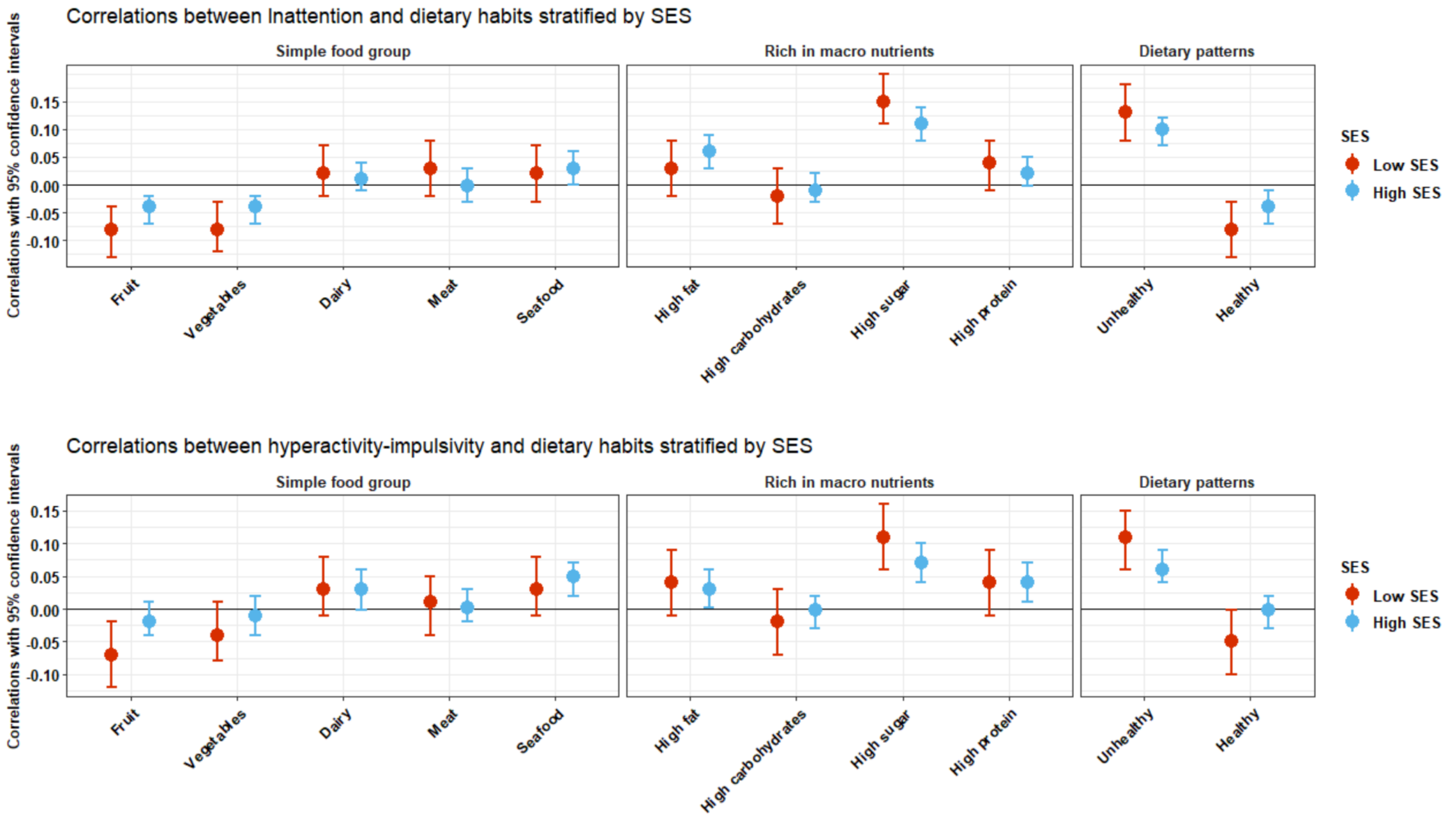

Figure 4. SES-specific associations (correlations with 95% confidence intervals) between ADHD trait dimensions and different dietary habits investigated in the primary analyses
